# Psychometric evaluation of the Problem Areas in Diabetes (PAID) survey in Southern, rural African American women with Type 2 diabetes

**DOI:** 10.1186/1471-2458-8-70

**Published:** 2008-02-22

**Authors:** Stephania T Miller, Tom A Elasy

**Affiliations:** 1 Department of Surgery, Center for Women's Health Research, Meharry Medical College, Nashville TN, USA; 2 Vanderbilt Eskind Diabetes Clinic and Center for Health Services Research, Vanderbilt University Medical Center, Nashville TN, USA

## Abstract

**Background:**

The Problem Areas in Diabetes (PAID) survey is a measure of diabetes-related stress for which reported use has been in largely Caucasian populations. Our purpose was to assess the psychometric properties of the PAID in Southern rural African American women with Type 2 diabetes.

**Methods:**

A convenience sample of African American women (N = 131) ranging from 21–50 years of age and diagnosed with Type 2 diabetes were recruited for a survey study from two rural Southern community health centers. Participants completed the PAID, Center for Epidemiological Studies-Depression Scale (CES-D), and the Summary of Diabetes Self-Care Activities Scale (SDSCA). Factor analysis, Cronbach's coefficient alpha, and construct validation facilitated psychometric evaluation.

**Results:**

A principle component factor analysis of the PAID yielded two factors, 1) a lack of confidence subscale, and 2) a negative emotional consequences subscale. The Lack of Confidence and Negative Emotional Consequences subscales, but not the overall PAID scale, were associated with glycemic control and body mass index, respectively. Relationships with measures of depression and diabetes self-care supported construct validity of both subscales. Both subscales had acceptable (alpha = 0.85 and 0.94) internal consistency measures.

**Conclusion:**

A psychometrically sound two-factor solution to the PAID survey is identified in Southern, rural African American women with Type 2 diabetes. Lack of confidence in and negative emotional consequences of diabetes self-care implementation provide a better understanding of determinants of glycemic control and weight than an aggregate of the two scales.

## Background

Type 2 diabetes is a leading cause of death for African American women [1]. Epidemiological evidence indicates that glycemic control in this patient group is suboptimal and that they suffer disproportionately from diabetes-related complications [2].

Though tight glycemic control is viewed as a primary indicator of favorable diabetes outcomes [3], a myriad of factors, including attention to diet, monitoring of blood glucose, medication, and physical activity [4], contribute to a patient's success in achieving desirable glycemic control. Additionally, psychological distress can negatively impact a patient's adherence to these necessary self-care regimens [5,6].

The Problem Areas in Diabetes (PAID) survey was developed as a measure of diabetes-related stress that could be useful in measuring the association between psychological adjustment to diabetes and adherence to self-care behaviors [7]. This 20-item survey uses a Likert-scale format to assess the degree to which diabetes management and/or feelings about diabetes are problematic to patients. To date, research application of the PAID has been mostly in Caucasian, urban populations [8,9] and, to a lesser extent, urban African Americans [1,10]. Our goal was to assess the reliability and validity of the PAID in Southern, rural African American women with Type 2 diabetes.

## Methods

### Subjects

This study was part of a larger survey investigation to characterize diabetes self-care activities in Southern, rural African American women with Type 2 diabetes. Participants were recruited from two rural community health centers in a southern state. Both centers serve predominately African American patients. Eligible participants were 1) African American females, 2) with a clinical diagnosis of Type 2 diabetes for a minimum of 6 months, and 3) between 21 and 50 years of age. Participants were identified from a diabetes database and sent invitation letters or referred by nurses during appointments. Follow-up phone calls were used when patients had not responded to letters after 2 weeks. Newspaper ads and flyers were also used for recruitment. The Meharry Medical College Institutional Review Board approved the survey and the administration procedures.

### Measures

Demographic characteristics were obtained using a self-report survey. Body mass index (BMI) was calculated from self-reported height and weight entries. The most recent hemoglobin A1c (HbA1c) readings within the last year were collected via chart extractions after all surveys were complete.

Problem Areas in Diabetes (PAID) scores were calculated using a five-point Likert-scale with options ranging from "0-not a problem" to "4-serious problem". Summing all item scores and multiplying by 1.25 resulted in an overall PAID score. A minimum score of 0 indicated no diabetes-related distress. A maximum score of 100 indicated significant diabetes-related distress. Previously, high internal consistency of PAID was reported (Cronbach's alpha = 0.95) and factor analyses revealed a single global emotional distress factor [11]. Evidence of construct validity has been reported based on correlations to related measures, including diabetes coping scales [11]. Additionally, discriminant validity has been reported, including comparisons of PAID scores between Type 1 and 2 diabetes patients [11].

The Center for Epidemiological Studies Depression Scale (CES-D) [8] was used as a measure of depression. Scores were based on patients' responses to 20 statements assessing behavior and feelings within the last week. Response options ranged from "0-rarely/none of the time" to "3-most/all of the time". Summing all item scores resulted in a final score, with a score of 60 indicating significant depression.

The Summary of Diabetes Self-Care Activities Scale (SDSCA) [12] was used to measure various diabetes self-care activities. Using a continuous scale ranging from "0 to 7 days", each participant indicated the number of days in a week that she engaged in 1) eating a generally healthy diet (general diet-2 items), 2) eating a diet high in fruits/vegetables and low in high fat foods (specific diet-2 items), 3) physical activity for at least 30 minutes (1 item), 4) a specific exercise (1 item), 5) glucose self-monitoring (2 items), 6) foot inspections (2 items), and 7) taking recommended medications (1 item). For self-care categories with at least 2 items (all except the "physical activity", "specific exercise", and "medication", questions), item scores were averaged resulting in an overall score for each self-care activity (i.e. general diet, specific diet, glucose self-monitoring, and foot inspections). Scores ranged from 0 (no weekly participation in a diabetes self-care activity) to 7 (participation in a diabetes self-care activity every day of the week).

### Implementation

All surveys were self-administered (paper and pencil) to individual patients or patient groups (ranging from 2 to 25 people). Administration took place in a designated room at the collaborating community health centers under the direct supervision of either the principal investigator or research coordinator. Patients received a $20 gift certificate for participation. Survey completion times ranged from 20 minutes to 40 minutes.

### Statistical Analysis

To examine the internal structure of the PAID, we performed principal component factor analysis to examine the pattern of loadings for evidence of a 1-factor solution that supports the current use of the PAID as a single scale [7] or the possibility of an alternate factor solution. We utilized both a scree plot and a minimum eigenvalue of 1.5 to guide extracting factors in subsequent analysis. Orthogonal and oblique rotations were explored to best approximate simple structure when extracting more than one factor. Item-loadings of 0.40 or greater were the criterion used to guide interpretation of rotated factor loadings. Reliability was assessed using Cronbach's coefficient alpha. For internal consistency testing, we accepted an alpha of ≥0.75. To evaluate the construct validity of the PAID, we performed several correlation analyses using the Pearson product-moment correlation coefficient (r). For correlation with the CES-D, we hypothesized that higher scores on the PAID (either as a one or two factor solution) would be positively associated (r = ~0.5) with higher scores on the CES-D. We hypothesized low to moderate (r = 0.10–0.30) negative correlations with SDSCA subscales. We further tested validity of the PAID using correlations between the PAID and patient age, BMI, and HbA1c and expected low to moderate (r = 0.10–0.30) correlations.

All data were analyzed using STATA Version 7 (STATA Corporation, College Station, TX).

## Results

Of the 160 patients that completed the survey, only 131 were included in the analysis. A total of 7 surveys were excluded because participants did not meet the age criterion. Twenty-two patients did not complete all 20 PAID survey items. Demographically, these patients were not different from those that completed all items. Table 1 provides a demographic and clinical profile of the 131 Southern, rural African American women that completed all PAID items. The profile depicts a middle age, obese, and low-income patient group with suboptimal glycemic control, the majority of which were non-smokers.

**Table 1:** Participant Characteristics

Age (years)	39.4 ± 8.2
Married (%)	23.7
Income greater than 20 K (%)	25.2
Some college or above (%)	47.7
Average duration of diabetes (years)	6.1.0 ± 5.6
Hemoglobin A1c	9 ± 2.4
BMI	35.5 ± 8.6
Insulin-Requiring (%)	36.9
Smoker (%)	17.7

Data are % or means ± standard deviations.

Internal consistency of the PAID was high (Cronbach's alpha = 0.94). Our factor analysis resulted in 2 factors with eigenvalues above 1.5 and with sound internal consistency (Factor 1 alpha = 0.85; Factor 2 alpha = 0.94). Orthogonal rotations best approximated simple structure for these 2 new factors. Factor loadings are presented in Table 2. Seven loaded on the first factor and 13 on the second factor. Items loading on the first factor were interpreted as a "Lack of Confidence in Self-Care Implementation" (Lack of Confidence Subscale) and those on the second factor as "Negative Emotional Consequences of Self-Care Implementation" (Emotional Consequences Subscale). We consequently elected to simultaneously investigate the construct validity of this 2-factor solution along with the single factor PAID solution.

**Table 2:** Item Loadings for Items in PAID Subscales

**PAID ITEMS**	Factor 1* Loadings-Lack of Confidence Subscale	**Factor 2† Loadings-Negative Emotional Consequences Subscale**
1. Not having clear and concrete goals for your diabetes care?	0.40	0.32
2. Feeling discouraged with your diabetes treatment plan?	0.73	0.31
3. Not "accepting" your diabetes?	0.66	0.39
4. Feeling unsatisfied with your diabetes physician?	0.61	0.07
5. Feeling that diabetes is taking up too much of your mental and physical energy everyday?	0.61	0.36
6. Feeling alone with your diabetes?	0.69	0.36
7. Feeling that your friends and family are not supportive of your diabetes management efforts?	0.65	0.05
8. Feeling scared when you think about living with diabetes?	0.22	0.78
9. Uncomfortable social situations related to your diabetes care (e.g., people telling you what to eat)?	0.49	0.49
10. Feelings of deprivation regarding food and meals?	0.39	0.46
11. Feeling depressed when you think about living with diabetes?	0.36	0.78
12. Not knowing if your mood or feelings are related to your diabetes	0.34	0.64
13. Feeling overwhelmed by your diabetes?	0.39	0.67
14. Worrying about low blood sugar reactions?	0.14	0.56
15. Feeling angry when you think about living with diabetes?	0.31	0.73
16. Feeling constantly concerned about food and eating?	0.18	0.81
17. Worrying about the future and the possibility of serious complications?	0.01	0.85
18. Feelings of guilt or anxiety when you get off track with your diabetes management?	0.35	0.65
19. Coping with complications of diabetes?	0.46	0.55
20. Feeling "burned out" by the constant effort needed to manage diabetes?	0.45	0.68

*First 7 items loaded on this factor (Lack of Confidence in Self-Care Implementation Subscale)

†Remaining 13 items loaded on this factor (Negative Emotional Consequences of Self-Care Implementation Subscale)

PAID = Problem Areas In Diabetes Scale

Correlations of the Lack of Confidence Subscale, Emotional Consequences Subscale, and the overall PAID to the CES-D (Cronbach's alpha = 0.85) and SDSCA subscales (Cronbach's alpha range = 0.75 to 0.87-for 2-item scales only) were used to assess construct validity based on a priori hypotheses (Table 3). The Cronbach's alpha estimate of the "specific diet" subscale was too low to establish internal consistency so we did not use this subscale in construct validity studies. This lack of internal consistency of this subscale has also been reported in studies of largely Caucasian populations [12]. Correlations were high (r = 0.69–0.97) between the Lack of Confidence subscale, Emotional Consequences subscale, and the PAID. In keeping with our hypothesis that depression would be positively correlated with the PAID, positive correlations were observed between the Lack of Confidence Subscale, Emotional Consequences Subscale, and the PAID and the CESD (r = 0.55–0.58). All correlations between the Lack of Confidence and Emotional Consequences subscales and SDSCA subscales were negative. This confirmed our theoretical assumption that diabetes-related stress would be higher for patients that participated in self-care activities less frequently. For the Lack of Confidence Subscale, significant negative correlations were found with diet and foot inspection-related subscales only. For the Emotional Consequences Subscale, significant negative correlations were observed with diet and physical activity-related subscales. With the exception of the glucose monitoring subscale and the medication adherence question, significant negative correlations were found between the PAID single factor solution and other SDSCA subscales.

**Table 3:** Pearson correlations between scores on the PAID, Lack of Confidence in Self-Care Implementation Subscale, and Emotional Consequences of Self-Care Implementation and scores on the CES-D, SDSCA subscales, and demographic and clinical factors.

	**PAID**	**Lack of Confidence in Self-Care Implementation**	**Emotional Consequences of Self-care Implementation**
**PAID**	1.0		
**Lack of Confidence in Self-Care Implementation**	0.83*	1.0	
**Emotional Consequences of Self-care Implementation**	0.97*	0.69*	1.0
**CES-D**	0.58*	0.58*	0.55*
**General Diet (SDSCA)-2 items**	-0.27*	0.30*	-0.23*
**Physical Activity (SDSCA)-1 item**	-0.26*	-0.20	-0.25*
**Exercise (SDSCA)-1 item**	-0.24*	-0.13	-0.27*
**Glucose Monitoring (SDSCA)-2 items**	-0.15	-0.12	-0.14
**Foot Inspections (SDSCA)-2 items**	-0.18*	-0.22*	-0.14
**Taking Medications-1 item**	-0.03	-0.10	-0.02
**Age**	-0.24*	-0.23*	-0.22*
**Hba1c**	0.21	0.27*	0.17
**BMI**	0.18	0.08	0.20*

*p ≤ 0.05

PAID = Problem Areas In Diabetes Scale; CES-D = Center for Epidemiological Studies Depression Scale; SDSca = Summary of Diabetes Self-Care Activities Scale

Correlations of the Lack of Confidence and Emotional Consequences subscales and the PAID with select demographic and clinical characteristics were used to examine predictive validity. Age was significantly negatively correlated with the each subscale and the PAID. Glycemic control, as measured by HbA1c, was significantly correlated (in a positive direction) with only the Lack of Confidence subscale. Body mass index was positively correlated with each of the subscales and the PAID. However, the association was only statistically significant with the Emotional Consequences subscale.

## Discussion

This is the first study to quantitatively evaluate the psychometric attributes of the highly utilized PAID survey in Southern, rural African American women with Type 2 diabetes. While the original 1-factor solution previously described performed well, a 2-factor solution revealed clearer correlations between psychological distress and glycemic control and BMI, two critical indicators for diabetes-related health outcomes.

**Figure 1:**
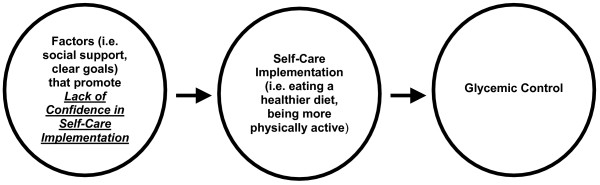
Relationship Between Lack of Confidence in Self-Care Implementation and Glycemic Control.

Importantly, we were able to observe a significant positive correlation between the 1st subscale in our 2-factor solution, "Lack of Confidence in Self-Care Implementation" and Hba1c. The fact that Hba1c was not significantly correlated with the PAID, as a single scale, is inconsistent with other findings ([7,9]) in largely Caucasian populations but consistent with findings in urban African Americans [10]. While the impact of race on PAID responses is not clear, it is possible that the PAID as a single scale, masks some of the more specific factors that impact emotions and behaviors that ultimately influence HbA1c in African American women. However, additional work is required to explore this notion. Figure 1 provides a conceptual model and shows that Lack of Confidence in implementing the diabetes self-care plan could potentially influence self-care, which might ultimately impact glycemic control. The clinical relevance of this finding is apparent, especially for this high-risk population, and is of critical importance in delineating relevant emotional factors that may influence self-care behaviors and, ultimately, impact glycemic control. This relationship between Lack of Confidence and glycemic control is consistent with the findings of others that confidence is a key determinant of self-care behaviors among African American women with Type 2 diabetes [13] and persons with Type 2 diabetes, in general [14]. Since there was not a significant correlation between the PAID and HbA1c, this finding also provides support for the additional utility of the PAID as 2-factor model.

**Figure 2:**
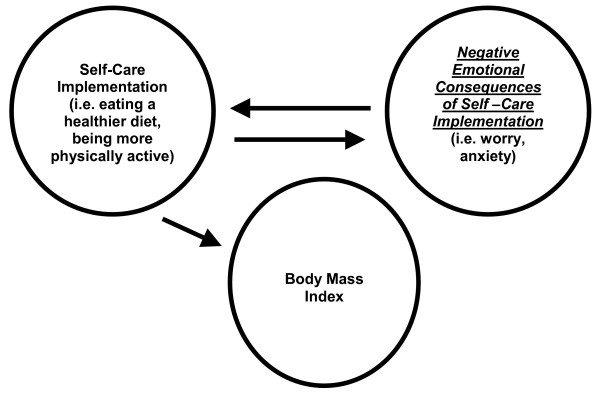
**Relationship Between Negative Emotional Consequences of Self-Care Implementation and Body Mass Index**.

Moreover, our 2-factor solution revealed a significant positive association between our 2^nd ^subscale, "Negative Emotional Consequences of Self-care Implementation" and BMI. This association is clinically relevant given the emerging obesity epidemic among African American women [15] and the disproportionate diabetes burden among African American women [2]. For example, items in the Emotional Consequences subscale represent the emotional consequences of implementing a diabetes self-care plan. Figure 2 illustrates this relationship. It shows that implementing a diabetes self-care plan could possibly result in negative emotions that might, in turn, impact self-care. Further, the degree to which specific components of the self-care plan are implemented can negatively influence BMI.

Therefore, our 2-factor solution allowed us to identify important correlates of HbA1c and BMI in Southern, rural African American women with Type 2 diabetes, ones that would not have emerged using the 1-factor PAID solution alone. Our work represents first steps in identifying relevant emotional factors for diabetes self-management and, ultimately, glycemic control among rural African American women with Type 2 diabetes. To the extent that our 2 subscales reliably and accurately correlate with Hba1C and BMI among this patient population, future work should focus on defining best methods for improving patient confidence in self-care activities that pose the most significant challenges and helping patients manage the emotional consequences of implementing self-care plans.

Our study has some limitations. Cognitive response interviews were not conducted. The addition of this method would have enhanced our ability to understand how patients perceived the meaning of individual PAID items. Our demographic questionnaire did not discriminate between patients receiving insulin via shots or pump. Therefore, our study results do not take into account differences attributable to distinct methods of insulin delivery and, possibly, adjustment issues. Another possible limitation was the potential for patients to respond to PAID questions in a manner that they felt was socially desirable (i.e. reporting that "feeling scared when you think about living with diabetes" was not a problem when indeed it was a significant one). Though impractical relative to minimizing patient fatigue during questionnaire administration, the additional administration of a social desirability questionnaire along with the PAID and other questionnaires would have been helpful in determining whether this was indeed true for this study [16]. Also, our most significant study results were found in a sample of obese patients with poorly controlled diabetes. Since our study only included rural, female patients, it is not clear the extent to which gender and locale alone correlated with these specific clinical characteristics. A more heterogeneous study sample would have been helpful in this regard. From a psychometric perspective, it was apparent from our factor analysis results that 3 of the PAID items loaded well (≥ 0.40) on both the Lack of Confidence and Emotional Consequences subscales. Since it was not our goal to modify the PAID items and, hence, possibly enhance factor loading clarity, we assigned these 3 items to the Emotional Consequences subscale based on three critical factors: 1) slightly superior loadings (with the exception of item 9 where loadings were equal); 2) face validity, and 3) better theoretical cohesiveness relative to our construct validity conceptualization. Finally, while it is appropriate and common practice to report correlations between 1- and 2-item SDSCA subscales and other scales, subscales with more items might lend to more robust and discriminating results.

The greatest strengths of our study were findings that lack of confidence in diabetes self-care implementation and the negative emotional consequences of self-care implementation were associated with Hba1c and BMI, respectively. Though the correlations that support these findings were modest, the clinical importance of identifying emotions that might impact glycemic control and body weight among Southern, rural African American women with Type 2 diabetes adds tremendous value to these novel findings. Other major strengths of this study were related to our recruitment efforts. We were able to recruit African American patients, a group that is typically perceived to be "hard to reach" relative to study recruitment ([17]). Additionally, we were able to recruit patients from rural locales, a success that is invaluable for involving geographically underserved populations in research ([18]).

## Conclusion

A psychometrically sound two-factor solution to the PAID survey is identified in Southern, rural African American women with Type 2 diabetes. Lack of confidence in and negative emotional consequences of diabetes self-care implementation provide a better understanding of determinants of glycemic control and weight than an aggregate of the two scales. Further work is needed to replicate these findings and, subsequently, design interventions that to improve the lives of Southern, rural African American women with Type 2 diabetes.

## Competing interests

The author(s) declare that they have no competing interest.

## Authors' contributions

STM was primarily responsible for the study design, implementation, data collection, data analysis, data interpretation, and drafting the manuscript. TAE provided detailed guidance on study design and implementation, aided substantially in data interpretation, and contributed to drafting and editing the manuscript.

## Pre-publication history

The pre-publication history for this paper can be accessed here:


